# The Relationship Between Serum Adiponectin, Tumor Necrosis Factor−Alpha, Leptin Levels and Insulin Sensitivity in Childhood and Adolescent Obesity: Adiponectin is a Marker of Metabolic Syndrome

**DOI:** 10.4274/jcrpe.v1i5.233

**Published:** 2009-08-04

**Authors:** Ayfer Alikaşifoğlu, E. Nazlı Gönç, Z. Alev Özön, Yaşar Şen, Nurgün Kandemir

**Affiliations:** 1 Pediatric Endocrinology Unit, Department of Pediatrics, Hacettepe University Faculty of Medicine, Ankara, Turkey; +90 312 305 18 88+90 312 312 18 09ayfera@hacettepe.edu.trPediatric Endocrinology Unit, Department of Pediatrics, Hacettepe University Faculty of Medicine, Ankara, Turkey

**Keywords:** children, obesity, Adiponectin, metabolic syndrome

## Abstract

**Objective**: This study aimed (a) to investigate the relationship between the degree of obesity and serum adiponectin, tumor necrosis factor (TNF)−α, leptin, insulin levels and the lipid profile; (b) to clarify the relationship between insulin resistance/glucose tolerance and adipocytokine levels; and (c) to investigate the value of adipocytokine levels as a marker of metabolic syndrome (MS).

**Methods**: We studied 151 obese children and adolescents (86 boys and 65 girls; mean age was 12.3±2.4 years). We defined obesity as a body−mass index (BMI) z−score more than 2 SD above the mean for age and sex. The control group consisted of 100 children (48 boys, 52 girls, mean age 12.4±2.5 years). Fasting glucose, insulin levels and lipid profiles were measured in all cases and controls after a 12−hour fast. Adiponectin, TNF−α, and leptin levels were measured in the subjects who participated in the adipocytokine branch of the study. An oral glucose tolerance test (OGTT) was also performed in all obese patients. Obese patients were grouped into three subgroups according to their glucose tolerance and insulin sensitivity assessment, and also according to whether they were grouped as MS or not.

**Results**: Serum levels of total cholesterol, LDL and VLDL cholesterol, log triglyceride, insulin, leptin and TNF−α were higher, whereas HDL and square root adiponectin levels were lower in the obese group when compared with controls. Multiple regression analysis among BMI−z score, LDL, triglyceride, HOMA−IR, leptin and TNF−α as determinants of adiponectin revealed that BMI−z score was the only determinant for adiponectin (r:−0.45, p<0.0001). Adiponectin levels in hyperinsulinemic and impaired glucose tolerance groups (IGT) tended to be lower than in normoinsulinemic obese children, however, the difference was not significant. There was a weak negative correlation between adiponectin levels and increasing severity of insulin resistance (r=−0.23, p=0.005) in the groups of obese subjects. Mean serum adiponectin level in subjects with MS was lower than in subjects without MS (p=0.008).

**Conflict of interest:**None declared.

## INTRODUCTION

Obesity and insulin resistance are on their way to become commonly encountered conditions both in childhood and adolescence. This is accompanied by an increase in the number of young obese populations with the metabolic syndrome (MS). The features of MS include insulin resistance, glucose intolerance, hypertension, dyslipidemia and central obesity. All these parameters are risk factors for coronary heart disease and type 2 diabetes mellitus (T2DM). Several new features, such as the decrease of adiponectin and the increase of plasminogen activator inhibitor−1 (PAI−1) and C−reactive protein (CRP) have been recently added to the definition of MS, which is considered as a low−grade inflammatory state. 

Adipose tissue is currently considered to be hormonally active and to take part in the control of metabolism. Adipose tissue secretes a large number of physiologically active peptides that have common properties with cytokines, and therefore referred to as adipocytokines. Leptin, tumor necrosis factor−alpha (TNF−α), PAI−1, interleukin−6 (IL−6) and adiponectin are some of these adipocytokines. Adiponectin is specifically expressed in human adipocytes. Plasma adiponectin concentration is decreased in individuals with obesity, and body−weight reduction increases its concentration (1, 2, 3, 4). Several studies have suggested that adiponectin may have a role in the modulation of glucose metabolism and insulin sensitivity (5, 6, 7, 8, 9, 10). Recent studies have demonstrated that administration of adiponectin to rodents increased insulin−induced tyrosine phosphorylation of the insulin receptor in skeletal muscle, resulting in improved glucose tolerance (11). It is also suggested that adiponectin has an anti−inflammatory effect on the vascular wall and also has an anti−atherosclerotic effect by direct action on the endothelial cells (12, 13). Clinical studies have shown that adiponectin levels are lower in individuals with coronary artery disease (14, 15). However, the relationships between adiponectin levels and atherosclerosis or metabolic abnormalities such as insulin resistance, are still obscure. 

The aim of this study was to analyze (a) the relationship between the degree of obesity and serum adiponectin, TNF−α, leptin, insulin levels, lipid profile; (b) the relationship between insulin resistance/glucose tolerance and adipocytokine levels; and (c) the value of adipocytokine level as a marker of MS.

## METHODS

**Patients and Controls**

We studied 151 obese children and adolescents (86 boys and 65 girls). The mean age of the patients was 12.3±2.4 years (range 7−17 years). We defined obesity as a body−mass index (BMI) z−score greater than 2 standard deviations above the mean for age and sex. Due to the age range in the study population z score was used for evaluation and a cutoff of 2 SD was taken instead of an absolute value of 30kg/m^2^. The control group consisted of 100 (48 boys, 52 girls) nonobese, healthy children and adolescents who presented to the outpatient clinic for acute problems or routine check−up. The mean age of the control group was 12.4±2.5 years (6−16.8 years).

Height was measured to the nearest millimeter by a wall−mounted stadiometer, and weight was measured to the nearest 100 g by SECA digital scale with light clothing. 

Fasting glucose, insulin levels and lipid profiles (total cholesterol, HDL, LDL, VLDL cholesterol, and triglycerides) were assessed in all cases and controls after a 12−hour fast. In obese subjects, thyroid function tests, diurnal cortisol levels, basal ACTH levels were also measured to exclude hypothyroidism or hypercortisolism. An oral glucose tolerance test (OGTT) was also performed in these subjects. 

Obese subjects and 29 controls (17 boys, 12 girls) agreed to participate in the adipocytokine branch of the study. The mean age of the 29 control cases was 12.7±2.6 years (range 7.6−18.2 years). This group was similar to the whole group with respect to age, sex distribution, pubertal status and BMI. Adiponectin, TNF−α, and leptin levels were measured in the subjects who participated in the adipocytokine assessment. 

The patients were not receiving any antihypertensive medication or drugs that can interfere with glucose or lipid metabolism at the time of the measurements.

**Calculations**

The following calculations were made using respective formulae: 

− Body−mass index (BMI) was calculated as: Weight (kg)/height^2^ (m). BMI z−score was calculated using the LMS method (16), according to the equation BMI z−score= (BMI/M)L−1/LS, where L represents the Box−Cox power transform required to remove the skewness from the distribution, M − the median of body mass index according to age, and S − the coefficient of variation. 

− HOMA−IR was calculated as: Fasting insulin (μU/ml) x fasting glucose (mmol/L) / 22.5. The cutoff point for insulin sensitivity was determined as 4.17 using the 95^th^ percentile of HOMA−IR in 100 control cases. Since prepubertal subjects comprised less than 15% of both obese and control groups and the distribution of pubertal stages was similar in both groups, this single cutoff level for HOMA−IR was used.

**Study Design**

The following analyses were performed:

a) The clinical and biochemical characteristics of the obese population were compared with healthy controls. 

b) The effect of degree of obesity as determined by BMI−z score on serum adipocytokine levels was analyzed by linear correlation. BMI−z score and other parameters (LDL, triglyceride, HOMA−IR, leptin and TNF−α which may affect adiponectin) were analyzed by multiple regression to find the determinants of adiponectin.

c) The association between insulin resistance/glucose tolerance and adipocytokine levels was analyzed. For this analysis obese patients were grouped into three subgroups according to glucose tolerance and insulin sensitivity. WHO criteria for glucose tolerance and our cutoff level for insulin resistance as determined by HOMA−IR (4, 17) were used to group the patients as follows: 

Group 1 (n=90): Normal glucose tolerance and normoinsulinemia (NGT + NI)

Group 2 (n=44): Normal glucose tolerance and hyperinsulinemia (NGT + HI)

Group 3 (n=17): Impaired glucose tolerance or type 2 diabetes mellitus (IGT or DM). 

Fifteen (9.9%) patients presented with impaired glucose tolerance (IGT) and two patients (1.3%) were diabetic. 

Leptin, TNF−α, and adiponectin levels of these groups were compared.

d) We also analyzed the value of adipocytokine level as a marker for MS. Obese patients were grouped according to the presence or absence of MS. We used the modified criteria of Viner et al (17) for the diagnosis of MS (modified from the National Cholesterol Education Program, Adult Treatment Panel). Dyslipidemia (triglyceride level >95^th^ percentile, HDL level <5^th^ percentile), elevated blood pressure (systolic or diastolic blood pressure > 95^th^ percentile), IGT and insulin resistance were the four criteria of MS. The patients were classified as having MS if they met 3 or more of these criteria for age and sex. Lipid Research Clinics Program (LRCP) levels were used to define dyslipidemia (18). Blood pressure nomograms for Turkish children and adolescents were used to define hypertension (19). Seventy two obese patients (47.6%) out of 151 were defined as having MS.

We compared adipocytokine levels of obese cases with and without MS. 

The study was approved by the Ethics Committee of our Institute, and informed consent was obtained from all subjects and their parents after a full explanation of the study.

**Assays**

Plasma glucose and lipid levels were measured by autoanalyzer (Roche Diagnostics). Plasma insulin levels were measured using immunoradiometric assay (Biosource INS−IRMA kit). Plasma adiponectin levels were measured by radioimmunoassay (Linco Laboratories). The intra− and inter−assay coefficients of variation were 4.5−7.6% and 3.9−6.6%. TNF−α was measured using enzyme−linked immunosorbent assay (ELISA) (Biosource). The intra− and inter−assay coefficients of variation were 3.9−5.2% and 5.9−8.5%. Leptin was measured using an immunoradiometric assay (IRMA) (Diagnostic System Laboratories, Inc.).

**Statistical Analysis**

The comparisons of normally distributed parameters were carried out using the student’s t test. We used logarithmic (log) transformation for several variables (triglyceride, HOMA−IR) since their distribution was skewed. Square root transformation was used to convert adiponectin values to normal distribution. We used Spearman’s correlation analysis, multiple regression, student’s t−test and ANOVA in the analyses. Statistics Package for Social Sciences (SPSS) version 13.0 was used for the statistical analyses. P values <0.05 were considered significant.

## RESULTS

**a) Comparison of the clinical and biochemical parameters of the study population with controls**

The clinical and biochemical characteristics of the study population are shown in Tables 1 and 2. Serum levels of total cholesterol, LDL and VLDL cholesterol, log triglyceride, insulin, leptin and TNF−α were higher (p<0.001; p<0.001; p<0.001; p<0.0001; p=0.005; p<0.001; p<0.0001, respectively), whereas HDL and square root adiponectin levels were lower (p<0.001) in the obese group when compared to the controls.

**b) The effect of degree of obesity on serum adipocytokine levels**

**Figure 1:**
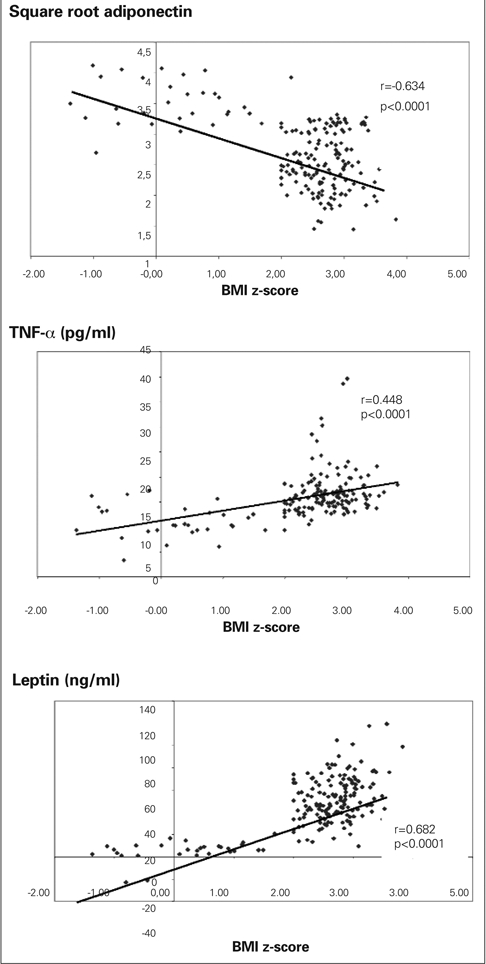
Relationship between adipocytokines and the degree of obesity (BMI z−score)

BMI z−score was positively correlated to TNF−α, leptin, log triglyceride, cholesterol, LDL, VLDL and log HOMA−IR (r=0.448, p<0.0001; r=0.682, p<0.0001; r=0.364, p<0.0001; r=0.243, p<0.001; r=0.267, p<0.0001, r=0.230, p=0.002; r=0.314, p<0.0001) and negatively correlated to the square root of adiponectin and HDL (r=−0.634, p <0.0001; r=− 0.281, p < 0.0001) (Figure 1).

Multiple regression analysis among BMI−z score, LDL, triglyceride, HOMA−IR, leptin and TNF−α as determinants of adiponectin revealed that BMI−z score was the only determinant for adiponectin (r=−0.45, p<0.0001).

**c) The association between insulin resistance/glucose tolerance and adipocytokine levels**

Leptin, adiponectin and TNF−α levels of obese subjects were significantly different than the controls. However, adipocytokine levels did not differ across groups of insulin sensitivity among obese subjects. There was a weak negative correlation between adiponectin levels against groups of obese subjects with increasing severity of insulin resistance (r=−0.23, p=0.005) (Table 3).

**d) The value of adipocytokine levels as a marker of MS**

The baseline metabolic characteristics of obese patients with MS are shown on Table 4. Mean serum adiponectin level in subjects with MS was 6.1±2.4 μg/ml, while this level was 7.2±2.5 μg/ml in obese subjects without MS. The difference between the two groups was statistically significant (p=0.008).

**Figure 2:**
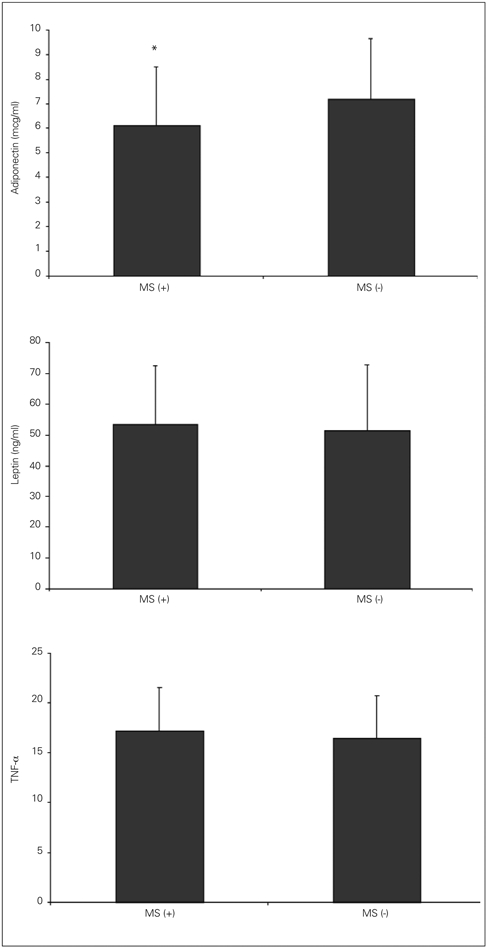
Adipocytokine levels of obese patients with (MS) in comparison to those without MS * p<0.01

The serum levels of leptin and TNF−α were comparable between the two groups (p=0.547 and p=0.281). (Figure 2).

**Tables 1:**
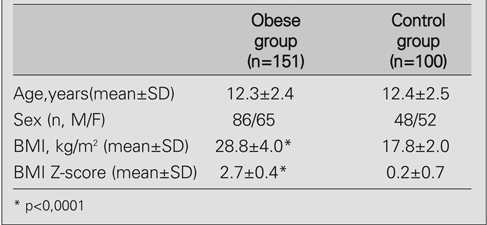
Characteristics of the study population

**2:**
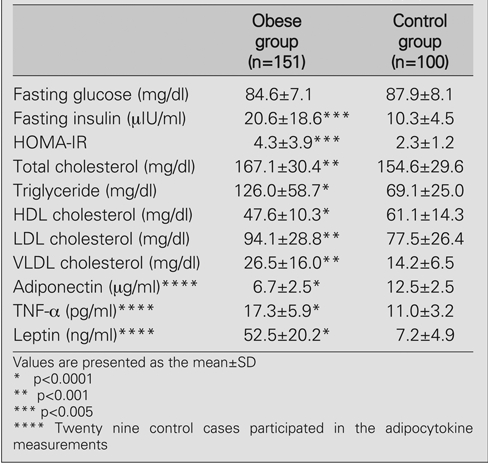
Baseline metabolic characteristics of the study population

**Table 3:**
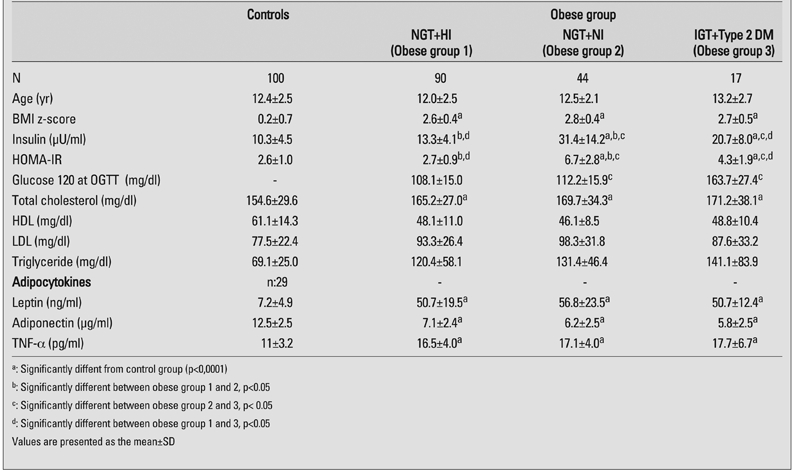
Metabolic profile based on level of insulin sensitivity

**Table 4:**
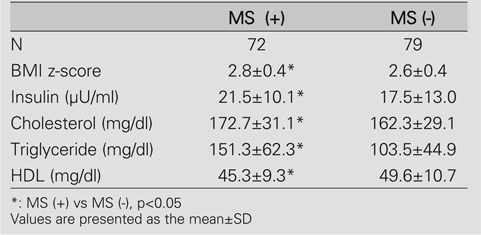
Baseline metabolic characteristics of obese patients with metabolic syndrome (MS) in comparison to those without MS

## DISCUSSION

The adipocytes are known to have an active endocrine function; IL−6, TNF−α, PAI−1, leptin and adiponectin are produced in adipose tissue (20). Leptin and adiponectin are the major adipocytokines secreted by fat cells. Leptin effects energy homeostasis by decreasing food intake and by acting on lipogenesis and fatty acid oxidation. In contrast to leptin, plasma adiponectin concentrations are inversely related to adiposity. Evidence suggests that adiponectin inhibits the production and action of TNF−α (21). TNF−α interferes with the insulin signaling cascade (21). Leptin and adiponectin modify glucose and lipid metabolism, insulin sensitivity, food intake, inflammatory processes and cardiovascular function. Although the physiological role of adiponectin has not yet been fully elucidated, this protein has been implicated in the pathophysiology of obesity−related insulin resistance, glucose intolerance, insulin−mediated lipoprotein metabolism, atherosclerosis, and coronary heart disease (1, 7, 8, 12, 13, 22). 

The mechanisms responsible for the control of adiponectin synthesis have not been determined so far. Insulin has been the only hormone implicated in the regulation of adiponectin expression. During a hyperinsulinemic−euglycemic glucose clamp, adiponectin levels were suppressed below basal levels in both diabetic and non−diabetic patients (23). Weight loss induces an increase in adiponectin levels in obese subjects. This state suggests the existence of a negative feedback mechanism between adipose mass and the production of adiponectin in humans (4). 

Studies in adults support the role of adiponectin in the pathogenesis of insulin resistance and T2DM (24, 25, 26). Several studies demonstrated the association between adiponectin and measures of insulin resistance also in the pediatric age group (9, 27, 28, 29, 30). Punthkee et al (31) showed that adiponectin was not independently associated with markers of insulin resistance in youth. In our study, adipocytokine levels did not differ within obese groups when they were grouped according to insulin resistance and glucose tolerance. Adiponectin levels in the groups with hyperinsulinemia and IGT tended to be lower than in normoinsulinemic obese children, however, the difference was not significant. Lack of statistical significance between adiponectin levels of obese children with different degrees of glucose tolerance does not necessarily mean that these two parameters are unrelated. The number of patients in each group in the current study may be too small for such a conclusion. Also, there are three isoforms of adiponectin in the serum: low, middle and high molecular weight. Recent studies showed that glucose tolerance is better correlated with the levels of high molecular weight (HMW) complexes in the serum rather than total adiponectin (32). HMW adiponectin might be a better biomarker of insulin resistance than the commonly used measure of total adiponectin. The mechanisms implicated in lower adiponectin levels in subjects with insulin resistance also remain obscure. TNF−α is one of the candidate molecules responsible for causing insulin resistance. The expression and secretion of adiponectin from adipocytes are significantly reduced by TNF−α. Therefore, increased TNF−α might be partially responsible for the decreased adiponectin production in obesity. 

In adults, epidemiological studies have shown that serum adiponectin levels are negatively correlated with various indices of MS such as hypertension, insulin resistance, glucose intolerance and dyslipidemia. In 967 Japanese adult subjects with normal weight, Yamamoto et al (26) have shown that plasma adiponectin is negatively correlated with BMI, systolic and diastolic blood pressure, fasting plasma glucose, insulin, insulin resistance, total and low−density lipoprotein−cholesterol, triglycerides and uric acid and positively correlated with high−density−lipoprotein cholesterol. Lower plasma adiponectin levels are observed not only among obese patients, but patients with T2DM and coronary artery disease also have reduced adiponectin levels. Hotta et al (8) showed that adiponectin levels in patients with T2DM were lower than those of non−diabetic patients, and were particularly low in subjects with coronary artery disease. The study by Weyer et al (24) demonstrated that hypoadiponectinemia was more intensively related to the degree of insulin resistance and hyperinsulinemia than to the degree of adiposity or glucose intolerance. Matsubara et al (33) have shown that plasma adiponectin is negatively correlated with serum triglyceride, atherogenic index, apo B, or apo E, and positively correlated with HDL−cholesterol or apo A−I levels. These data suggest that low adiponectin concentrations are associated with some of the well known risk factors for atherosclerosis such as low HDL−cholesterol levels or hypertriglyceridemia. It seems likely that a relationship exists between hypoadiponectinemia and MS. 

An inverse correlation between serum adiponectin levels and hyperinsulinemia, insulin resistance and dyslipidemia has also been reported in the limited number of studies conducted in children (3, 10, 28, 29, 31, 34). Winer et al (35) performed a standard OGTT and obtained baseline measurements for adiponectin, plasma lipid profile, CRP, IL−6 and leptin in a multiethnic cohort of 589 obese children and adolescents. These authors found that the link between adiponectin levels and a strong marker of inflammation, CRP, is independent of insulin resistance and adiposity in obese children and adolescents. They suggested that adiponectin may have a function as a biomarker of MS in childhood obesity. Ogawa et al (36) have shown that hypoadiponectinemia was associated with visceral fat accumulation and MS in obese boys. Our findings are consistent with previous reports suggesting that serum adiponectin levels may serve as a marker for MS (36, 37, 38, 39, 40). 

In conclusion, among the adipocytokines evaluated in the present study, adiponectin is the best indicator of MS and we believe that the assessment of adiponectin levels might contribute to early intervention in obese children with MS.
